# Patient satisfaction and associated factors among insured and uninsured patients in Deder General Hospital, eastern Ethiopia: a facility-based comparative cross-sectional study

**DOI:** 10.3389/fmed.2023.1259840

**Published:** 2023-12-27

**Authors:** Giduma Shure, Mulugeta Gamachu, Habtamu Mitiku, Alemayehu Deressa, Addis Eyeberu, Fethia Mohammed, Hamdi Fekredin Zakaria, Galana Mamo Ayana, Abdi Birhanu, Adera Debella, Ibsa Mussa

**Affiliations:** ^1^Goro Muti Woreda Health Office, East Hararghe, Ethiopia; ^2^School of Medicine, College of Health and Medical Sciences, Haramaya University, Harar, Ethiopia; ^3^Department of Public Health, Rift Valley University, Harar, Ethiopia; ^4^School of Medical Laboratory Sciences, College of Health and Medical Sciences, Haramaya University, Harar, Ethiopia; ^5^School of Public Health, College of Health and Medical Sciences, Haramaya University, Harar, Ethiopia; ^6^School of Nursing and Midwifery, College of Health and Medical Sciences, Haramaya University, Harar, Ethiopia; ^7^Department of Psychiatry, School of Nursing and Midwifery, College of Health and Medical Sciences, Haramaya University, Harar, Ethiopia

**Keywords:** insured, uninsured, satisfaction, patients, Deder hospital, eastern Ethiopia

## Abstract

**Background:**

Patient satisfaction is a crucial measure of healthcare quality, as dissatisfied patients are more likely to miss appointments, disregard treatment plans, and leave hospitals, leading to poor treatment outcomes. Therefore, the study aimed to compare levels of satisfaction with health services and associated factors among insured and uninsured patients in Deder General Hospital, eastern Ethiopia.

**Methods:**

A comparative cross-sectional study with 532 participants was conducted from December 1–30, 2021. Data was collected through a structured questionnaire, analyzed using SPSS, and predictors assessed using a multivariate logistic regression model. Statistical significance was set at *p* < 0.05.

**Results:**

Overall, patient satisfaction with health services was 65.6% (95% CI: 61.5–69.5), and the level of patient satisfaction with health services among insured and noninsured patients was 68.8% (95% CI: 62.8–74.4) and 62.4% (95% CI: 56.8–68.0), respectively. In the final model of multivariable analysis, factors such as educational status of secondary school (AOR = 4.90; 95% CI: 2.05–11.76), and a higher level (AOR = 3.08; 95% CI: 1.05–9.03), getting the entire prescribed drugs (AOR = 3.49; 95% CI: 1.43–8.54), getting some of the ordered drugs (AOR = 3.34; 95% CI: 1.61–6.94), paying less than 100 Ethiopian birrs (AOR = 4.85; 1.35–17.40) were significantly associated with patient satisfaction among insured patients. Whereas getting the entire and some prescribed drugs were (AOR = 6.28; 95% CI: 3.26–12.05), and (AOR = 3.40; 95% CI: 1.70–6.78) times more likely to be satisfied with the service among noninsured patients as compared to their counterparts, respectively.

**Conclusion:**

The study found that about six in 10 patients in the study area were satisfied with healthcare services, with insurance patients reporting higher satisfaction. Factors such as receiving prescribed drugs, paying less than 100 Ethiopian birr, having a secondary school education, and having a higher education were associated with satisfaction.

## Introduction

The world has had more access to essential health services in recent years than at any other time in human history. In 2017, African health ministers agreed to strengthen health systems through interventions, aiming to achieve Universal Health Coverage (UHC) in Sub-Saharan African countries, despite regional disparities and resource shortages, and despite increased access to essential health services (1). UHC is a situation where “all people can access the health services they need without incurring financial hardship” (2). Ethiopia’s public health sector is promoting financial protection through social health insurance and community-based health insurance, aiming to secure financial protection for over 85% of the informal sector (3). The Ethiopian Federal Ministry of Health has developed a strategic plan (2016–2020) to achieve quality health services in the whole country (4). The strategic plan aims for comprehensive, safe, effective, patient-centered healthcare in Ethiopia, delivered efficiently, affordable, and measured by patient satisfaction (4, 5).

Patient satisfaction is an essential component of patient-centered care, and it plays a significant role in the healthcare delivery system (6). It is an important and commonly used indicator for measuring healthcare quality that affects clinical outcomes, patient retention, and medical malpractice claims and affects the timely, efficient, and patient-centered delivery of quality healthcare (7). Patient satisfaction comprises three fundamental characteristics of a framework for enhancing health system performance: increasing the patient experience of care, improving the health of populations, and reducing the *per capita* cost of healthcare (8, 9).

Over the last decades, patients’ perceptions of health care have become an important indicator for measuring the quality of health care (10). Poor treatment outcomes are frequently caused by dissatisfied patients, who are more likely to skip visits, ignore treatment regimens, and leave hospitals against medical staff advice (11, 12). Also, it can increase anxiety and irritability in patients, which results in delayed recovery time, and more beds in the hospital will be occupied by increasing the length of hospitalization and costs of treatment (13).

Different studies conducted in different developed and developing countries show an insignificant level of patient satisfaction (6, 14–18). In this regard, several initiatives and plans have been made by various interested bodies on the organizational, healthcare provider, and client sides to enhance and preserve patient satisfaction (19, 20). The Ethiopian Health Sector Reform (EHSR) aims to increase the efficacy, efficiency, and caliber of services delivered by health facilities (4). In addition, Ethiopian administrations have developed a UHC approach that aims to provide financial protection through community-based health insurance (CBHI) programs to achieve patient satisfaction and quality as per the standards (3, 21). This CBHI scheme began in four dominant regions of the ruling party at that time in 2011 as pilot schemes in the 13 Woredas of Amhara; Oromia; Southern Nations Nationalities and Peoples (SNNP); and Tigray regional states (3). Deder General Hospital was one of these pilot sites in Oromia; however, patient satisfaction among patients with insurance and those without insurance has not been measured since 2011.

Despite the tremendous benefit of maintaining patient satisfaction, research from several sources revealed that most patients prefer to visit private facilities over public ones because they are less satisfied with the quality of medical care provided (22, 23). Indeed, the experience of care does play a role in quality, and devoting resources toward improving patients’ experiences and ultimately satisfaction will benefit both the patient and the hospital (24). In addition to delivering and tracking its change over time, routine measuring of patient satisfaction levels for improvement is the recommended approach for healthcare providers at all levels (21). Therefore, the current study aimed to compare levels of satisfaction with health services and associated factors among insured and uninsured patients in Deder General Hospital, eastern Ethiopia.

## Methodology

### Study design, setting and period

An institution-based comparative cross-sectional facility-based study was conducted from December 1–30, 2021 among those who are insured and non-insured under the CBHI scheme at Deder General Hospital, East Hararghe Zone, Oromia, Ethiopia. Deder Hospital is one of the eight hospitals’ in the East Hararghe zone and serves four populated woredas’ in East Hararghe (Malka Ballo, Goro Gutu, and Deder Woreda). According to the 2016 EDHS report, the hospital was serving about 357,904 males and 344,128 females, for a total of 702,032 populations being served by the hospital. Deder General Hospital was the first health facility where the federal government began a pilot study of community-based health insurance in 2011.

### Study design

An institution-based comparative cross-sectional study was implemented.

### Population and eligibility criteria

All insured and noninsured patients who visited the outpatient department of the hospital during the study period were considered a source population. Whereas, all patients selected by a systematic random sampling method from outpatients who visited the hospital in the study period were the study populations. All insured and non-insured patients aged greater than 18 who visited the outpatient department of the hospital were included in the study, whereas patients who were critically ill and unable to communicate were not included in the study.

### Sample size determination and sampling procedures

The sample size for the study was determined for both objectives by the double population formula using EPI Info version 7, and the largest number was selected. The sample size was calculated using the power formula of 80%.


$$n=\frac{p1\left(1-p1\right)+p2\left(1-p2\right)}{\left(p1-p2\right)2}\times \left(\alpha ,\beta \right)$$

The assumption were.

n = sample size.

P1 = proportion of satisfaction on availability of drugs in insured patients was taken as 60%, and P2 = proportion of satisfaction on availability of drugs in non-insured patients was taken as 72% from the study conducted in public hospitals in Addis Ababa, Ethiopia (25).

α = the level of significance (0.05).

β = Type II error 0.2; and none response rate 10%.

Depending on the above statistical assumption, the final sample size calculated for this study was.

n= $\frac{0.60\left(1-0.60\right)+0.72\left(1-0.72\right)}{\left(0.60-0.72\right)2}x7.9$ =532.

**Figure 1:**
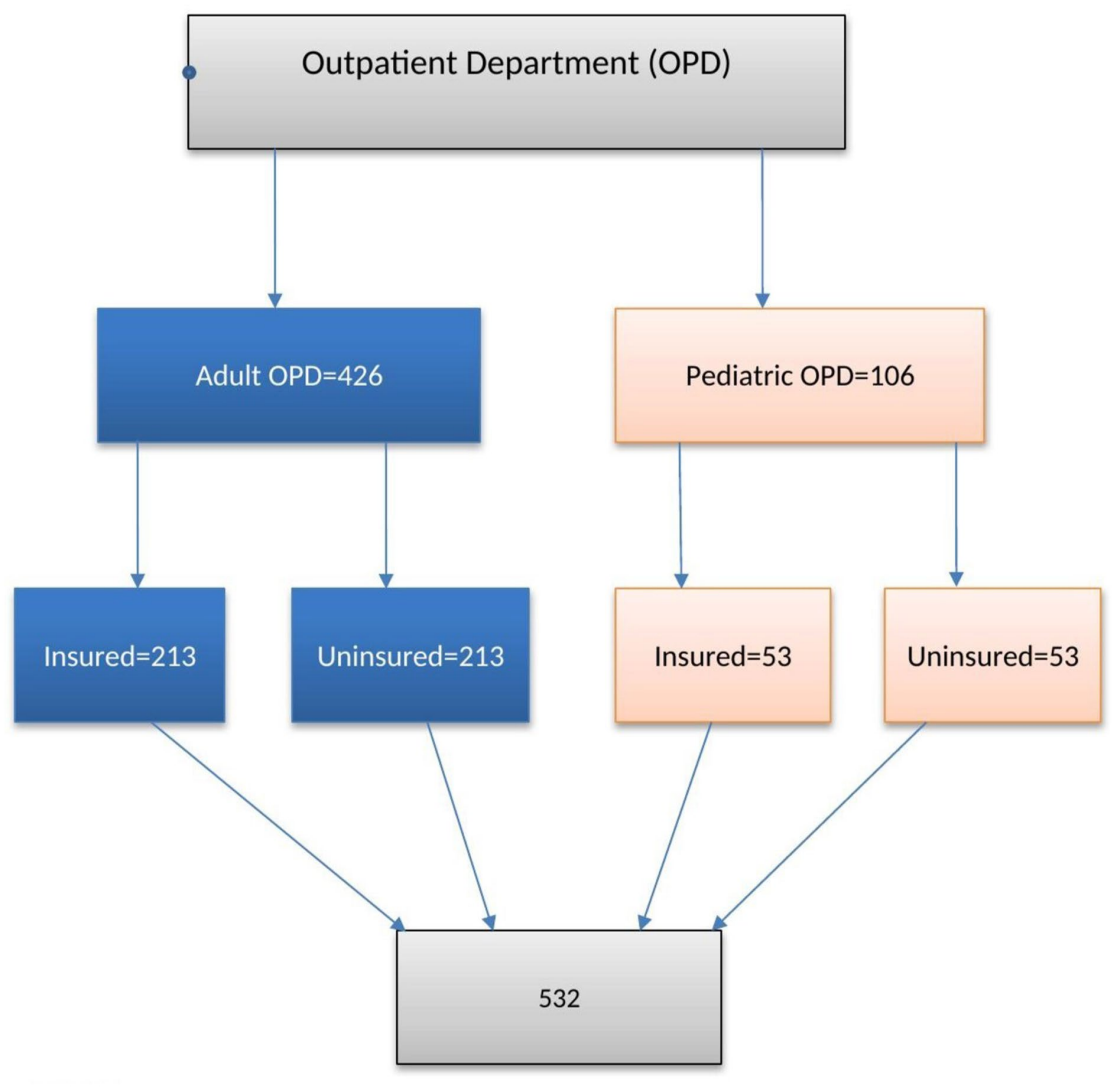
Sampling procedure to assess level of patient satisfaction on health services and associated factors among insured and non-insured patients in Deder Hospital Ethiopia.

Therefore, the final sample size for the study was 532, and a one-to-one allocation method was used for both groups (insured and non-insured). Finally, the factor that gives the largest sample size was used to determine the final desired sample size for the study. Therefore, the study used a sample size of 266 for insured and non-insured patients. The study subjects were selected from the adult and pediatric outpatient departments of the hospital using systematic random sampling using the patient’s identification number (Figure 1).

### Data collection methods

A structured, interviewer-administered questionnaire, extracted from various pieces of literature, was used for data collection. The outcome variable of the study was patient satisfaction with health services. The independent variables of the study were socio-demographic characteristics (age, sex, marital status, educational levels, religion, and income). Health services-related factors (waiting times, availability of prescribed drugs, laboratory services, cost of services, staff services, privacy and confidentiality, information, accessibility, provider’s behavior and services, physical facilities, overall satisfaction with the quality of care received from the facility), and consultation and diagnosis (weighing the patient, measuring the temperature, using a stethoscope, examining the patient, asking about a history of past illness, asking about a history of present illness, asking if treatment was taken before arrival at the facility, and explaining the diagnosis).

### Operational definition and measurements

Community-based health insurance (CBHI) is a prepayment method of financial contributions for healthcare that aims at risk pooling and the avoidance of catastrophic and impoverishing health expenditures (26).

Patient expectations were measured by the patient’s expectations, wants, and thoughts about what needed to be completed (25).

Not getting services: if the patient perceives he or she did not get all of the services (27).

Satisfaction with health services was measured using 16 questionnaires of five-point Likert scale items each rated from 1 (strongly disagree) to 5 (strongly agree), and the mean score was computed from the composite index score created from a summed score of 16 items. The level of satisfaction was satisfied when the participants scored above the mean score of the summed satisfaction index score and poor unless otherwise stated (28).

Insured Patient: A patient or their guardians who has paid an annual premium voluntarily to a local health care fund within a given period (2020/2021) and who had accessed care in the hospital at the time of interview.

A non-insured patient is a patient or caregiver who paid the full cost of each visit to the hospital but who had not accessed care in the hospital at the time of the interview.

Outpatient Department (OPD): the unit in the hospital where medical services are provided to clients.

Waiting time: the time interval between departures from registration for outpatient service and being seen by a physician at OPD.

### Data collection procedure

The data was collected using an interviewer-administered structured questionnaire that was developed after a review of different literature that supports the objectives of the study (27, 29, 30). An exit interview data collection method was performed. The questionnaire, which consists of the Likert type, was first prepared in English, then translated into the native language (Afaan Oromo) for data collection purposes, and then back-translated to English to make sure the meaning was retained. The data was collected by two BSc nurses and two diploma clinical nurse professionals who were trained on the principles of data collection, components of tools, and ethical issues. Respondents were asked about their perceived satisfaction with the hospital services they received on the day of the interview, and their responses were scored using a five-level Likert scale of categories (very good =5, good = 4, somewhat good = 3, poor = 2, and very poor = 1).

### Data quality control

The questionnaire was pretested on 5% of the sample size before the actual data collection at Chelenko Hospital, which provides similar services to the current study setting. Then the appropriateness of the checklist was evaluated, and vague questions were modified before the actual data collection. Besides, supervisors and the primary investigator regularly evaluated the obtained data for consistency, clarity, accuracy, and completeness. The internal reliability of 16 Likert scale perceived quality of health services measurement tools was estimated using Cronbach’s alpha value of 0.919, which was higher than the accepted value of 0.80 recommended by an author Kline (30).

### Data processing and analysis

All data were checked for completeness, cleaned, coded, and entered into Epi-data version 4.6 and exported to SPSS version 22.0 for further analysis. Descriptive statistics were used to summarize the data by using simple frequency tables and figures. The respondents were asked about their perceived satisfaction with the health care services they received and scored using a five-level Likert scale of categories. To examine the presence of a statistically significant association between insured and noninsured patients, a chi-square test was performed. Binary logistic regression was used to identify factors associated with satisfaction. All variables that were significant at a *p* value <0.25 in the bivariable analysis were considered for the multivariable analysis to control for all possible confounders. The model’s fitness was checked by the Hosmer-Lemeshow tests, and it was at a *p*-value of 0.314. Odd ratios along with the 95% CI were estimated to measure the strength of factors associated with the level of satisfaction with health services in both insured and non-insured patients. For all variables, the level of statistical significance was declared at *p* values less than 0.05.

### Ethical considerations

Ethical approval was obtained from the Institutional Health Research Ethics Review Committee (IHRERC) of Haramaya University, College of Health and Medical Sciences. Then formal letters of cooperation were taken from East Hararghe zonal health offices to Deder General Hospital. Before obtaining informed consent from patients and guardians, they were given a clear description of the study objectives, procedure, duration, and possible risks and benefits of the study. Their right to participate or not and to withdraw in the middle of the interview was guaranteed. Informed, written, and signed consent was obtained from voluntary patients (guardians). The confidentiality of all study participants was maintained.

## Results

### Socio-demographic characteristics of the participants

A total of 532 (266 insured and 266 uninsured) patients participated in this study, with a response rate of 100%. The total insured study participants, about half, 269 (50.6%), were female. Individuals who were insured and those who were not insured had a mean age with standard deviation (SD) of 43.10 ± 14.64 and 35.86 ± 13.39 years, respectively. Besides, seventy-four (27.8%) insured patients and sixty-six (24.8%) uninsured patients were aged equal to or greater than 50 years old. More than half of the study participants were urban dwellers 144 (54.1%) insured and 152 (57.1%) uninsured patients. Most of the study participants, insured 133 (50%) and uninsured 120 (45.1%), were Muslims. Regarding educational status, 39 (14.7%) of the respondents in the insured population and 45 (16.9%) uninsured patients attended higher educational levels, respectively. More than half of the study participants were urban dwellers, with 144 (54.1%) insured and 152 (57.1%) uninsured patients (Table 1).

**Table 1:** Socio-demographic characteristics of insured and uninsured patients in Deder General Hospital, Oromia region, eastern Ethiopia, 2021 (*n* = 532).

Variables	Categories	Insured (*n* = 266)	Uninsured (*n* = 266)	Total (%)
		Frequency (%)	Frequency (%)	
Sex	Male	132 (49.6)	131 (49.2)	263 (49.4)
	Female	134 (50.4)	135 (50.8)	269 (50.6)
Age	18–28	67 (25.2)	76 (28.6)	143 (26.9)
	29–39	73 (27.5)	67 (25.2)	140 (26.3)
	40–50	52 (19.5)	57 (21.4)	109 (20.5)
	>50	74 (27.8)	66 (24.8)	140 (26.3)
Marital	Single	65 (24.4)	71 (26.7)	136 (25.6)
	Married	150 (56.4)	156 (58.6)	306 (57.5)
	Divorced	33 (12.4)	25 (9.40)	58 (10.9)
	Widowed	18 (6.8)	14 (5.2)	32 (6.0)
Occupation	Student	59 (22.2)	42 (15.80)	101 (19.0)
	Farmer	77 (28.9)	73 (27.4)	150 (28.1)
	Merchant	54 (20.3)	68 (25.6)	122 (23.0)
	Others	76 (28.6)	83 (31.2)	159 (29.9)
Religion	Muslim	133 (50.0)	120 (45.1)	253 (47.6)
	Orthodox	56 (21.0)	69 (25.9)	125 (23.5)
	Protestant	47 (17.7)	51 (19.2)	98 (18.4)
	Others	30 (11.3)	26 (9.8)	56 (10.5)
Educations	No formal School	84 (31.6)	43 (16.2)	127 (23.9)
	Elementary School	83 (31.2)	112 (42.1)	195 (36.7)
	Secondary School	60 (22.5)	66 (24.8)	126 (23.6)
	Higher School	39 (14.7)	45 (16.9)	84 (15.8)
Residence	Urban	144 (54.1)	152 (57.1)	296 (55.7)
	Rural	122 (45.9)	114 (42.9)	236 (44.4)
Income	<=1,200	155 (58.3)	104 (39.1)	259 (48.7)
	>1,200	111 (41.7)	162 (60.9)	273(51.3)

*Other = daily laborer, and no jobs, **others = Catholic and Wakefata.

### Health services related factors

Out of all the participants in this study, only 98 (36.8%) insured and 149 (56.0%) uninsured patients reported that pharmacy staff gave explanations on the use of medications. In similar circumstances, 219 (82.3%) insured and 200 (75.2%) uninsured patients were reported as having their privacy and confidentiality maintained. Out of all the participants, only 81 (30.4%) of the insured and 84 (31.6%) of the uninsured patients received all of the prescribed medication. Similarly, only 32 (12.0%) of insured patients and fifty-seven (21.4%) of uninsured patients were seen by doctors within 30 min after arrival. Regarding the cost of services, 38 (14.3%) of insured patients and 47 (17.7%) of uninsured patients paid less than 100 Ethiopian birr (Table 2).

**Table 2:** Health services related factors among insured and uninsured patient in Deder General Hospital, Oromia region, eastern Ethiopia, 2021 (*n* = 532).

Variables	Categories	Insured (*n* = 266)	Uninsured (*n* = 266)
		Frequency (%)	Frequency (%)
Environment convenient to ask question(s)	No	42 (15.8)	66 (24.8)
	Yes	224 (84.2)	200 (75.2)
Pharmacy staff explained use of drugs	No	168 (63.2)	117 (44.0)
	Yes	98 (36.8)	149 (56.0)
Privacy and confidentiality kept	No	47 (17.7)	66 (24.8)
	Yes	219 (82.3)	200 (75.2)
Availability of ordered drugs	Not at all	121 (45.5)	88 (33.1)
	Some	64 (24.1)	94 (35.3)
	All in all	81 (30.4)	84 (31.6)
Availability ordered laboratory	Not at all	39 (14.7)	40 (15.0)
	Some	66 (24.8)	73 (27.5)
	All in all	161 (60.5)	153 (57.5)
Waiting time in minute	>90	108 (40.6)	55 (20.7)
	60–90	63 (23.7)	69 (25.9)
	30–60	63 (23.7)	85 (32.0)
	<30	32 (12.0)	57 (21.4)
Cost of services in birr	>500	0 (0.0)	144 (54.1)
	100–500	228 (85.7)	75 (28.2)
	<100	38 (14.3)	47 (17.7)

### Consultations and diagnosis

Regarding the objective quality of care measures used for consultations and diagnosis, 90 (33.8%) of insured and 136 (51.1%) uninsured patients, 84 (31.6%) of insured and 128 (48.1%) of uninsured patients, and 86 (32.3%) of insured and 104 (39.1%) of uninsured patients reported that providers measured the temperature of the patient, used a stethoscope, and weighed the patients, respectively. The diagnosis was explained only for 95 (35.7%) of insured patients and 143 (53.8%) of uninsured patients who were seen in outpatient services. Only 97 (36.5%) insured and 144 (54.1%) uninsured patients were asked if the treatment was taken before arrival (Table 3).

**Table 3:** Consultation and diagnostic care among insured and Uninsured patients attending Deder General Hospital, Oromia region, eastern Ethiopia, 2021 (*n* = 532).

Consultation and diagnosis factors	Categories	Insured (*n* = 266)	Uninsured (*n* = 266)
		Frequency (%)	Frequency (%)
Provider measure temperature of the patient	No	176 (66.2)	130 (48.9)
	Yes	90 (33.8)	136 (51.1)
Did the provider use a stethoscope?	No	182 (68.4)	138 (51.9)
	Yes	84 (31.6)	128 (48.1)
Provider examine head to toe	No	205 (77.1)	163 (61.3)
	Yes	61 (22.9)	103 (38.7)
Provider weigh the patient	No	180 (67.7)	162 (60.9)
	Yes	86 (32.3)	104 (39.1)
Asked history of present illness	No	14 (5.3)	11 (4.1)
	Yes	252 (94.7)	255 (95.9)
Did the provider ask about history of past illness?	No	173 (65.0)	135 (50.8)
	Yes	93 (35.0)	131 (49.2)
Did the provider explain the diagnosis to the patient	No	171 (64.3)	123 (46.2)
	Yes	95 (35.7)	143 (53.8)
Did the provider ask if treatment was taken before arrival at facility	No	169 (63.5)	122 (45.9)
	Yes	97 (36.5)	144 (54.1)

### Level of patient satisfaction with the CBHI scheme

**Figure 2:**
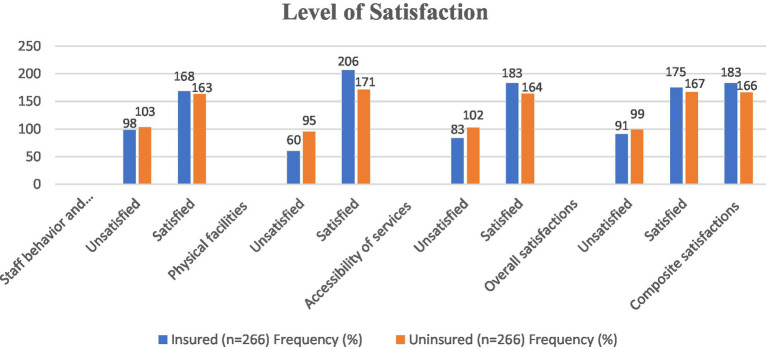
Factors affecting beneficiaries’ satisfaction with the CBHI Scheme in Deder General Hospital, Oromia region, eastern Ethiopia (*n* = 532), 2021.

The level of household satisfaction with the CBHI scheme was rated using five questions, each worth five points on a Likert scale. The CBHI plan satisfaction score ranged from 5 to a maximum of 25 points for respondents. The average level of satisfaction was 4.178. After that, families were classified as satisfied if their score was higher than the mean and unsatisfied if it was below the mean. Overall patient satisfaction at Deder General Hospital was 65.6% (95% CI: 61.5–69.50) based on 349 out of 532 respondents rating the hospital’s medical services as satisfactory. Overall, 183 (68.8%; 95% CI: 62.8–74.4) insured patients and 166 (62.4%; 95% CI: 56.8–68.0) uninsured patients expressed pleasure (Figure 2).

### Factors associated with patient satisfaction in insured

In the bi-variable model, factors such as education level, how patients visited the hospital, use of a stethoscope, measure of temperature, how patients visited the hospital, waiting time, whether pharmacy staff explained the use of drugs or not, availability of drugs, and cost of service were significant at a 0.25 value of p and considered for the final multivariable analysis to assess factors associated with satisfaction among insured patients. In the final multivariable analysis, three variables (level of education, availability of drugs, and cost of service) were found to have a statistically significant association with satisfaction.

The odds of being satisfied with health care services were 4.90 (AOR = 4.90; 95% CI: 2.05–11.75) and 3.08 (AOR = 3.08; 95% CI: 1.05–9.03) times higher among patients who attended secondary school and had a higher level of education compared to individuals without a formal education. Similarly, patients who received all of the prescription medication were 3.49 (AOR: 3.49; 95% CI: 1.43–8.54) times more satisfied than those who received only a portion of the ordered medication, who were 3.34 (AOR: 3.34; 95% CI: 1.61–6.94) times more satisfied than those who received none of the prescribed medication at all. Moreover, an insured patient who paid less than 100 Ethiopian birr for treatment and follow-up services was 4.85 (AOR: 4.85; 1.35–17.40) times more satisfied than those who were paid between 100 and 500 Ethiopian birr and those treated with an annual payment (Table 4).

**Table 4:** Bi-variable and multi-variable logistic regression analysis of factors associated with patient satisfaction in an insured at Deder General Hospital, Oromia region, eastern Ethiopia, 2021 (*n* = 266).

Variables	Categories	Satisfaction status of insured		COR (95% CI)	AOR (95% CI)
		Satisfied (%)	Not satisfied (%)		
Educations	No formal School	47 (56.0)	37 (44.0)	1	1
	Elementary School	54 (65.1)	29 (34.9)	1.43 (0.78–2.64)	1.76 (0.87–3.56)
	Secondary School	49 (81.7)	11 (18.3)	3.27 (1.62–6.59)	4.90 (2.05–11.75)*
	Higher School	23 (59.0)	16 (41.0)	7.70 (2.91–20.35)	3.08 (1.05–9.03)*
How to come to hospital	Self	65 (69.1)	29 (30.9)	1	1
	Recommendations	17 (56.7)	13 (43.3)	0.56 (0.24–1.29)	0.82 (0.32–2.07)
	Referral	101 (71.1)	41 (28.9)	1.42 (0.83–2.40)	0.83 (0.43–1.58)
Provider measure temperature	No	112 (63.6)	64 (36.4)	1	1
	Yes	70 (77.8)	20 (22.2)	1.95 (1.09–3.50)	1.70 (0.87–3.31)
Provider use a Stethoscope	No	116 (63.7)	66 (36.3)	1	1
	Yes	67 (79.8)	17 (20.2)	2.24 (1.25–4.14)	2.09 (1.13–3.86)
Pharmacy staff explained use of drugs	No	110 (65.5)	58 (34.5)	1	1
	Yes	73 (74.5)	25 (25.5)	1.59 (0.96–2.67)	1.32 (0.69–2.49)
Availability of ordered drugs	Not at all	76 (62.8)	45 (37.2)	1	1
	Some	50 (78.1)	14 (21.9)	3.93 (2.06–7.69)	3.34 (1.61–6.94)*
	All in all	49 (60.5)	32 (39.5)	5.16 (2.26–11.74)	3.49 (1.43–8.54)*
Waiting time in minute	>90	58 (53.7)	50 (46.3)		1
	60–90	33 (52.4)	30 (47.6)	0.95 (0.51–1.77)	0.84 (0.39–1.81)
	30–60	39 (61.9)	24 (38.1)	1.40 (0.74–2.64)	1.62 (0.75–3.49)
	<30	22 (68.7)	10 (31.3)	1.89 (0.82–4.38)	1.81 (0.67–4.89)
Cost of services in birr	100–500	148 (64.9)	80 (35.1)	1	1
	<100	35 (92.1)	3 (7.9)	6.04 (2.28–16.04)	4.85 (1.35–17.40)*

*statistically significant.

### Factors associated with patient satisfaction in uninsured

Factors such as education level, use of a stethoscope, measuring of temperature, how patients visited the hospital, waiting time, whether pharmacy staff explained the use of drugs or not, availability of prescribed drugs, and amount of payment were candidates for multivariable analysis at a *p*-value less than 0.25 in bivariable mode. In multivariable analysis, only the availability of prescribed drugs was significantly associated with satisfaction in uninsured patients. Patients without insurance who received all of their doctor’s prescriptions were 6.28 times (AOR = 6.28; 95% CI: 3.26–12.05) more satisfied than those who received part of their prescriptions, and 3.40 times (AOR = 3.40; 95% CI: 1.70–6.78) more satisfied than those who did not receive all of their prescriptions (Table 5).

**Table 5:** Bi-variable and multi-variable logistic regression analysis of factors associated with patient satisfaction in uninsured at Deder General Hospital, Oromia region, eastern Ethiopia, 2021 (*n* = 266).

Variables	Categories	Satisfaction status of uninsured		COR (95% CI)	AOR (95% CI)
		Satisfied (%)	Not satisfied (%)		
Educations	No formal School	23 (53.5)	20 (46.5)	1	1
	Elementary School	62 (55.4)	50 (44.6)	1.08 (0.53–2.18)	0.78 (0.34–1.74)
	Secondary School	20 (30.3)	46 (69.7)	2.00 (0.90–4.45)	1.41 (0.58–3.42)
	Higher School	35 (77.8)	10 (22.2)	3.04 (1.20–7.66)	1.909 (0.69–5.25)
How to come to hospital	Self	71 (56.8)	54 (43.2)	1	1
	Recommendations	35 (70.0)	15 (30.0)	0.85 (0.42–1.73)	1.03 (0.42–2.54)
	Referral	60 (65.9)	31 (34.1)	1.68 (0.95–2.95)	1.72 (0.90–3.29)
Provider measure temperature	No	74 (56.9)	56 (43.1)		1
	Yes	82 (60.3)	54 (39.7)	2.55 (1.51–4.19)	1.42 (0.80–2.53)
Provider use a stethoscope	No	77 (55.8)	61 (44.2)	1	1
	Yes	89 (69.5)	39 (30.5)	2.53 (1.52–4.28)	1.29 (0.71–2.23)
Pharmacy staff explained use of drugs	No	68 (58.1)	49 (41.9)		1
	Yes	98 (65.8)	51 (34.2)	1.44 (0.87–2.37)	1.32 (0.75–2.33)
Availability of ordered drugs	Not at all	34 (38.6)	54 (61.4)		1
	Some	58 (61.7)	36 (38.3)	3.93 (2.01–7.69)	3.40 (1.70–6.78)*
	All in all	55 (65.5)	29 (34.5)	5.15 (2.26–11.74)	6.28 (3.26–12.05)*
Waiting time in minute	>90	30 (54.5)	25 (45.5)	1	1
	60–90	40 (58.0)	29 (42.0)	1.15 (0.56–2.35)	1.10 (0.49–2.42)
	30–60	60 (70.6)	25 (29.4)	2.12 (0.99–4.05)	1.60 (0.73–3.51)
	<30	36 (63.2)	21 (36.8)	1.43 (0.67–3.04)	1.19 (0.55–2.77)
Cost of services	>500	76 (52.8)	68 (47.2)	1	1
	100–500	51 (68.0)	24 (32.0)	1.57 (0.88–2.79)	1.99 (0.96–4.30)
	<100	37 (78.7)	10 (21.3)	1.97 (1.01–3.83)	1.97 (0.92–4.21)

*statistically significant.

## Discussion

This study was conducted to assess patient satisfaction and associated factors among insured and non-insured patients in Deder General Hospital, Eastern Ethiopia. According to this study, we found that 65.6% (95% CI: 61.5–69.50) of patients were overall satisfied with the health care services and pointed out that patients with insurance have higher levels of satisfaction (68.8%) than patients without insurance (62.4%). The final model of multivariable logistic regression analysis indicated that factors such as educational status, availability of ordered drugs, cost of services, and availability of ordered drugs were significantly associated with patient satisfaction among insured and non-insured patients, respectively.

The findings of this study show that overall patient satisfaction with healthcare services was 65.6%. The findings of this study are in harmony with studies done in Arsi, Ethiopia (63.4%) (31), north-central Nigeria (63.1%) (32), and southeastern Nigeria (68.8%) (25). However, the findings of this study were higher than those of studies conducted in Nigeria (58.1%) (25), Ethiopia (54.7%) and (54.1%) (33, 34), Turkey (53.3%) (35), Ghana (43%) (36), and southern Nigeria (40%) (37). A possible explanation may be a difference in sample size and socio-demographic characteristics.

Our findings also revealed that the satisfaction level among insured patients was 68.8%, while it was 62.4% among uninsured patients. This finding is lower than studies conducted in Northern Nigeria (25), Nepal (83.2% insured and uninsured patients, 84.8%) (38), Tanzania (71% insured and 81% uninsured patients) (28), North East Nigeria (76.5% insured patients and 68.7% uninsured patients) (25), and Ethiopia (79.4% insured and 75.7% uninsured patients) (39). The variation in sample size, socioeconomic level, and study design could all be contributing factors to the reason. It is a known fact that people in higher socioeconomic positions receive quality healthcare services, which leads to higher levels of satisfaction. According to a South African study, socioeconomic status is a key factor in determining how satisfied patients are with their access to healthcare services (40). Another possible explanation may be the availability of better health facilities that enhance the level of satisfaction.

This study found a strong correlation between the availability of necessary medications and patients’ satisfaction levels with the quality of healthcare services, both for insured and uninsured individuals. Patients who received all of the prescribed medications among insured and uninsured patients reported 3.49 and 6.28 times higher levels of satisfaction than those who received none, respectively. Investigations carried out in Nigeria (25), Ghana (41), Ethiopia (39), Northeast Nigeria (25), and Wollo Ethiopia (42) corroborate this conclusion. The insured patients who frequently visit their facility and are aware that drug absence is widespread in Ethiopian healthcare facilities due to a variety of circumstances may have a possible justification. Patients with insurance can then tolerate drug absences. The results of the investigation bear this out, revealing that only 30. 5% of insured patients obtained all of the prescription drugs (they start treatment with a roughly 69.5% drug shortfall in their dispensing room). Other investigations conducted in the eastern portion of Ethiopia (35.14%) (43) and in Arsi, Ethiopia (23.1%) (31) also support this finding. Therefore, the study implied that it is imperative to work on material and drug supply to increase patient satisfaction.

We found that the cost of services was significantly associated with the level of satisfaction among insureds. Insured patients who paid a manageable cost (less than 100 birr) were satisfied with 4.85 times more than those unsatisfied with the cost of services, but no significant association was seen with uninsured patients. It is in line with studies in northern Nigeria (25), Burkina Faso (44), Ghana (45), and northern Ethiopia (39). One explanation might be that the majority of the insured patients received government assistance, which included yearly insurance coverage. So, it decreases the financial gap between rich and poor people. It is seen practically that those relatively better off in the economy want proper treatment rather than suffer from the cost of treatment. It looks like this is why the cost of services has no significant association with uninsured patient satisfaction. This finding implies that strengthening health insurance will enhance satisfaction with health care services.

In this study, insured patients who had secondary and higher education status were 4.90 and 3.08 times more likely to be satisfied than those who had no formal education, respectively. Additionally, education levels have not shown a significant association with uninsured patients. The current finding was consistent with the study conducted in Arsi, Ethiopia (31), Nigeria (37), Addis Abeba (27), and Nekemte, Ethiopia (29). This finding is also in disagreement with the study done in the northern part of Ethiopia, where increased education levels reduce satisfaction in noninsured patients (39). Higher education may facilitate access to more information regarding medical procedures, which is one rationale that could be given. It can be difficult to fast-track those initiatives in communities with lower levels of education when new policies and procedures are implemented. This is a result of a misunderstanding about how new programs and regulations relate to other occurrences. As the majority of Ethiopians lack formal education, it is crucial to inform the community about new policies and procedures, particularly those relating to insurance programs.

### Limitation of the study

It may be challenging to provide more meaningful information regarding participant perception and the time sequence of the associations because the study only used a quantitative method and a cross-sectional design.

## Conclusion

Overall, 65.6% of patients were satisfied with health care services at Deder General Hospital, and a slightly higher level of satisfaction was observed among insured patients. However, there was no statistically significant difference observed in the level of satisfaction among insured and uninsured patients. Educational status, availability of prescribed drugs, and cost of services were found to have a statistically significant association with satisfaction in insured patients, while only the availability of prescribed drugs was a statistically significant factor that affected the satisfaction of uninsured patients. Therefore, program managers and health care providers should ensure the quality of services to meet standards at the health facility for insured and non-insured community members to improve patient experiences at health facilities and accomplish financial risk protection through CBHI. Additionally, hospital administrators must guarantee the availability of crucial medications to lessen patient dissatisfaction among both insured and uninsured patients.

## Data availability statement

The datasets used for this study are available from the corresponding authors upon reasonable request.

## Ethics statement

The studies involving humans were approved by Institutional Health Research Ethics Review Committee. The studies were conducted in accordance with the local legislation and institutional requirements. The participants provided their written informed consent to participate in this study. The manuscript presents research on animals that do not require ethical approval for their study.

## Author contributions

GS: Conceptualization, Data curation, Formal analysis, Investigation, Methodology, Software, Writing – original draft, Writing – review & editing. MG: Data curation, Investigation, Methodology, Software, Writing – review & editing. HM: Conceptualization, Data curation, Formal analysis, Investigation, Methodology, Software, Supervision, Validation, Writing – review & editing. AD: Conceptualization, Data curation, Formal analysis, Investigation, Methodology, Validation, Writing – review & editing. AE: Data curation, Investigation, Methodology, Software, Writing – review & editing. FM: Writing – review & editing. HZ: Data curation, Methodology, Writing – review & editing. GA: Data curation, Methodology, Writing – review & editing. AB: Writing – review & editing. AD: Data curation, Investigation, Methodology, Software, Writing – review & editing. IM: Conceptualization, Data curation, Formal analysis, Investigation, Methodology, Project administration, Software, Supervision, Validation, Writing – review & editing.
